# Liberal transfusion strategy to prevent mortality and anaemia-associated, ischaemic events in elderly non-cardiac surgical patients – the study design of the LIBERAL-Trial

**DOI:** 10.1186/s13063-019-3200-3

**Published:** 2019-02-04

**Authors:** Patrick Meybohm, Simone Lindau, Sascha Treskatsch, Roland Francis, Claudia Spies, Markus Velten, Maria Wittmann, Erdem Gueresir, Christian Stoppe, Ana Kowark, Mark Coburn, Sixten Selleng, Marcel Baschin, Gregor Jenichen, Melanie Meersch, Thomas Ermert, Alexander Zarbock, Peter Kranke, Markus Kredel, Antonia Helf, Rita Laufenberg-Feldmann, Marion Ferner, Eva Wittenmeier, Karl-Heinz Gürtler, Peter Kienbaum, Marcel Gama de Abreu, Michael Sander, Michael Bauer, Timo Seyfried, Matthias Gruenewald, Suma Choorapoikayil, Markus M. Mueller, Erhard Seifried, Oana Brosteanu, Holger Bogatsch, Dirk Hasenclever, Kai Zacharowski, LIBERAL Collaboration Group, David Baron, Oliver Grottke, Aileen Hill, Julia Van Waesberghe, Sebastian Ziemann, Markus Tingart, Julius van Essen, Oliver Spring, Raphael Pirzer, Ulrich Jaschinski, Axel R. Heller, Martin Ertmer, Elke Falk, Philipp Pickerodt, Alexander Schiemann, Katrin Schmidt, Sascha Tafelski, Ralf-Felix Trauzeddel, Carsten Perka, Heide Ehrentraut, Louise Fingehut, Andreas Hopf, Vera Guttenthaler, Claudia Neumann, Isabelle Osberghaus, Patrick Schuss, Anja Winkler, Hendrik Kohlhof, Dieter C. Wirtz, Robert Kalb, Jonas Hinterberg, Maximilian Schäfer, Thea Koch, Florian Piekarski, Linda Tanner, Kira Berg, Carolin Wiedenbeck, Isabell Maushagen, Daniela Zeisset, Christoph Fuellenbach, Sabine Westphal, Sophia Pfeiffer, Andreas Schnitzbauer, Ingo Marzi, Andreas Becker, Shahram Ghanaati, Thomas Schmitz-Rixen, Verena Brixner, Christof Geisen, Eva Herrmann, Fabian Edinger, Christian Koch, Christian Kunzemann, Dominik Leicht, Melanie Markmann, Sophie Ruhrmann, Emanuel Schneck, Dargmar Schulte, Matthias Gründling, Iris Brenig, Manuela Gerber, Kathleen Selleng, Sandra Wodrig, Frank Bloos, Petra Bloos, Anja Haucke, Karina Knuhr-Kohlberg, Steffi Kolanos, Katrin Schwope, Daniel Thomas, Corinna Buchholz, Birte Heller, Jochen Renner, Nina Schulz-Ruhtenberg, Gunnar Elke, Susanne Fischer, Sabine Hofbauer, Peter Straub, Sophie Zimmer, Mira Küllmar, Nadine Rosenow, Christina Massoth, Raphael Weiss, Diane Bitzinger, Karin Pfister, Ines Guzman, Eva-Maria Kranke, Norbert Roewer, Achim Woeckel, Philipp Helmer

**Affiliations:** 1 Department of Anaesthesiology, Intensive Care Medicine and Pain Therapy, University Hospital Frankfurt, Theodor-Stern-Kai 7, 60590 Frankfurt am Main, Germany; 2 Department of Anesthesiology, Operative Intensive Care Medicine, Berlin Institute of Health, Charité – Universitätsmedizin Berlin, Freie Universität Berlin, Humboldt-Universität zu Berlin, Campus Charite Mitte, Campus Virchow Klinikum, Campus Benjamin Franklin, Charité , Berlin, Germany; 3 Department of Anaesthesiology and Intensive Care Medicine, University Hospital Bonn, Bonn, Germany; 4 Department of Neurosurgery, University Hospital Bonn, Bonn, Germany; 5 Department of Intensive Care Medicine, RWTH University Aachen, Pauwelstrasse 30, 52074 Aachen, Germany; 6 Department of Anesthesiology, RWTH University Aachen, Pauwelstrasse 30, 52074 Aachen, Germany; 7 Department of Anesthesiology, University Hospital Greifswald, Greifswald, Germany; 8 Department of Anaesthesiology, Intensive Care and Pain Therapy, University Hospital Münster, Münster, Germany; 9 Department of Anaesthesia and Critical Care, University Hospital of Würzburg, Wuerzburg, Germany; 10 Department of Anaesthesiology and Intensive Care Medicine, University Hospital Mainz, Main, Germany; 11 Department of Anaesthesiology and Intensive Care Medicine, University Hospital Augsburg, Augsburg, Germany; 12 Department of Anaesthesiology and Intensive Care Medicine, University Hospital Düsseldorf, Düsseldorf, Germany; 13 Department of Anaesthesiology and Intensive Care Medicine, University Hospital Dresden, Dresden, Germany; 14 Department of Anaesthesiology and Intensive Care Medicine, University Hospital Giessen-Marburg, Giessen, Germany; 15 Department of Anaesthesiology and Intensive Care Medicine, University Hospital Jena, Jena, Germany; 16 Department of Anaesthesiology and Intensive Care Medicine, University Hospital Regensburg, Regensburg, Germany; 17 Department of Anaesthesiology and Intensive Care Medicine, University Hospital Schleswig-Holstein, Campus Kiel, Kiel, Germany; 18 Institute for Transfusion Medicine and Immunohaematology Frankfurt/ Main, German Red Cross Blood Transfusion Service Baden-Wuerttemberg – Hessen, Frankfurt, Germany; 19 Clinical Trial Centre Leipzig, University Leipzig, Leipzig, Germany; 20 Institute for Medical Informatics, Statistics and Epidemiology, University Leipzig, Leipzig, Germany

**Keywords:** Red blood cell transfusion, anaemia, surgery, elderly patients

## Abstract

**Background:**

Perioperative anaemia leads to impaired oxygen supply with a risk of vital organ ischaemia. In healthy and fit individuals, anaemia can be compensated by several mechanisms. Elderly patients, however, have less compensatory mechanisms because of multiple co-morbidities and age-related decline of functional reserves. The purpose of the study is to evaluate whether elderly surgical patients may benefit from a liberal red blood cell (RBC) transfusion strategy compared to a restrictive transfusion strategy.

**Methods:**

The LIBERAL Trial is a prospective, randomized, multicentre, controlled clinical phase IV trial randomising 2470 elderly (≥ 70 years) patients undergoing intermediate- or high-risk non-cardiac surgery. Registered patients will be randomised only if Haemoglobin (Hb) reaches ≤9 g/dl during surgery or within 3 days after surgery either to the LIBERAL group (transfusion of a single RBC unit when Hb ≤ 9 g/dl with a target range for the post-transfusion Hb level of 9–10.5 g/dl) or the RESTRICTIVE group (transfusion of a single RBC unit when Hb ≤ 7.5 g/dl with a target range for the post-transfusion Hb level of 7.5–9 g/dl). The intervention per patient will be followed until hospital discharge or up to 30 days after surgery, whichever occurs first. The primary efficacy outcome is defined as a composite of all-cause mortality, acute myocardial infarction, acute ischaemic stroke, acute kidney injury (stage III), acute mesenteric ischaemia and acute peripheral vascular ischaemia within 90 days after surgery. Infections requiring iv antibiotics with re-hospitalisation are assessed as important secondary endpoint. The primary endpoint will be analysed by logistic regression adjusting for age, cancer surgery (y/n), type of surgery (intermediate- or high-risk), and incorporating centres as random effect.

**Discussion:**

The LIBERAL-Trial will evaluate whether a liberal transfusion strategy reduces the occurrence of major adverse events after non-cardiac surgery in the geriatric population compared to a restrictive strategy within 90 days after surgery.

**Trial registration:**

ClinicalTrials.gov (identifier: NCT03369210).

**Electronic supplementary material:**

The online version of this article (10.1186/s13063-019-3200-3) contains supplementary material, which is available to authorized users.

## Background

Perioperative anaemia leads to impaired oxygen supply with a risk of vital organ ischaemia resulting in major events such as myocardial infarction, ischaemic stroke, acute kidney injury, or acute mesenteric ischaemia. In healthy and fit individuals, perioperative anaemia can be compensated by several mechanisms that preserve oxygen transport and oxygen delivery. Therefore, current guidelines recommend a restrictive red blood cell (RBC) transfusion strategy in non-bleeding asymptomatic stable patients [1, 2].

Several trials revealed that restrictive transfusion is as safe as compared to a liberal strategy. It is noteworthy that the majority of trials only included a limited proportion of elderly patients [3–7]. The compensatory mechanisms, however, are impaired in old and frail patients. Elderly patients have an increased prevalence of cardiovascular comorbidities and decline of functional reserves. In 70 year old patients, e.g. arterial hypertension is present in 75% [8], diabetes mellitus in 25% [9], and atrial fibrillation in 10% [10]. Therefore, normal anaemia-related compensatory mechanisms are severely impaired in elderly patients, which may result in greater vulnerability to anaemia-related ischaemic events and perioperative complications [11, 12].

Carson et al. studied 110 patients with acute coronary syndrome with a mean age of 71 years and found fewer major cardiac events and deaths if red blood cell (RBC) transfusion increased Hb > 10 g/dl compared to a restrictive strategy (10.9% vs. 25.5%) [13]. One small trial including 40 patients with hip fracture compared a liberal (RBC transfusion if Hb < 10 g/dl) and a restrictive group (if Hb < 8 g/dl), and demonstrated a 2.5-times higher 30-day mortality in the restrictive group [14]. The same group performed a subsequent trial enrolling 2016 patients older than 50 years of age, who had either a history of or risk factors for cardiovascular disease, and whose Hb level were < 10 g/dl after hip-fracture surgery. A restrictive strategy (Hb < 8 g/dl) was not superior to a liberal transfusion strategy (Hb < 10 g/dl) regarding mortality rate or inability to walk on 60-days follow-up [15].

Until now, the available evidence for transfusion criteria is not sufficient for elderly patients. Thus, it is unclear whether current practice and guidelines apply in a geriatric population [16]. Accordingly, major uncertainties exist among clinicians, and current clinical practice is variable [17, 18]. As a result of demographic changes clinicians have to handle a large number of elderly surgical patients with perioperative anaemia and relevant need for RBC transfusion. More than 50% of all RBC transfusions are used in elderly patients in daily practice, and current population dynamics will lead to an increasing demand for RBC transfusions in the old-age patient group [19].

We hypothesise that a liberal transfusion strategy reduces the occurrence of major adverse events defined as the composite of all-cause mortality and severe ischaemic events within 90 days in elderly patients undergoing non-cardiac surgery compared to a restrictive transfusion strategy.

## Methods

The LIBERAL-Trial is a prospective, randomized, open, multicentre, controlled clinical phase IV trial targeting a sample size of 2470 elderly surgical patients in 15–20 German sites.

### Study population

**Fig. 1:**
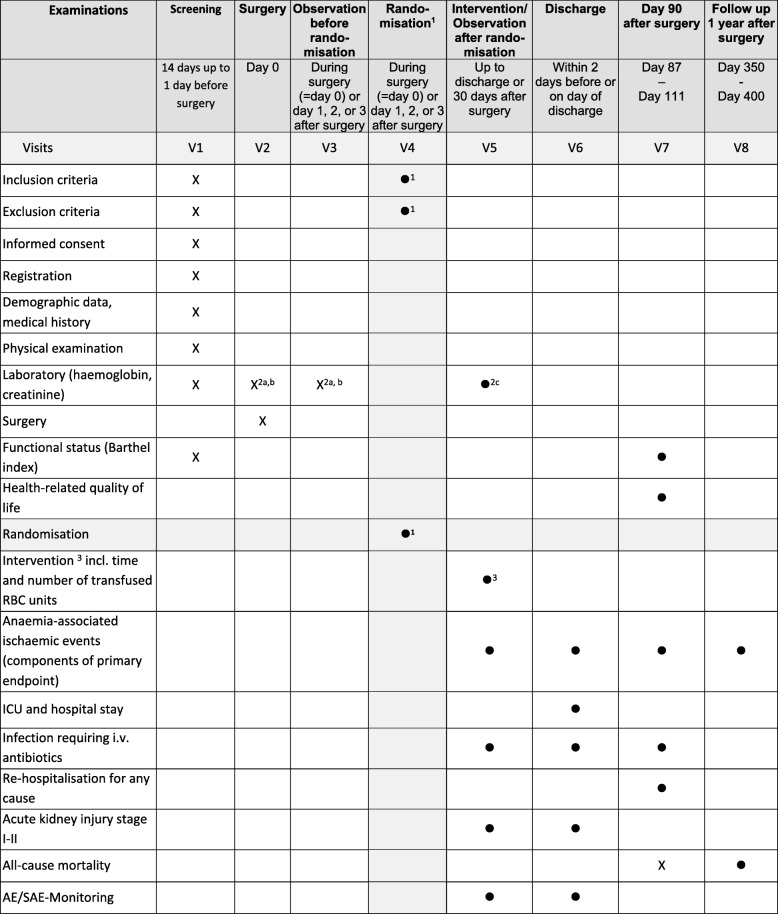
Schedule of Assessments and Procedures. X: assessments for all registered patients / •: additional assessments for randomised patients. ^1^ Randomisation: as soon as haemoglobin ≤9 g/dl during surgery (=day 0) or day 1, 2, or 3 after surgery, registered consenting patients will be randomised. Re-evaluation of inclusion−/exclusion criteria before randomisation only refers to obvious occurrence of any component of the composite endpoint and any allogeneic blood transfusion after registration (chapter 4.2.2.). No specified diagnostics are scheduled. ^2a^ Haemoglobin levels will be determined from blood samples (primarily BGA measurement mainly as part of the patient’s usual care) at least daily before randomisation. ^2b^ Mild drop of haemoglobin < 9 g/dl due to induction of anaesthesia before skin incision is permitted and not an exclusion criteria. ^2c^ Haemoglobin levels will be determined from blood samples (primarily BGA measurement mainly as part of the patient’s usual care) at any time during or after randomisation until hospital discharge (up to 30 days after surgery; at least every 3 days), and after each transfused unit. Creatinine levels will be determined as part of the patient’s usual care at any time during or after randomisation until hospital discharge (or up to 30 days after surgery; at least every 7 days). ^3^ Intra-/Postoperative Intervention: Duration of intervention per patient: from intra−/postoperative randomisation until hospital discharge or 30 days after surgery, whichever occurs first. Physicians will be instructed to transfuse RBC units each time haemoglobin is lower than the randomised threshold and as soon as possible. The randomised target post-transfusion Hb level needs to be reached each time within 24 h upon receipt of lab result at the latest

**Fig. 2:**
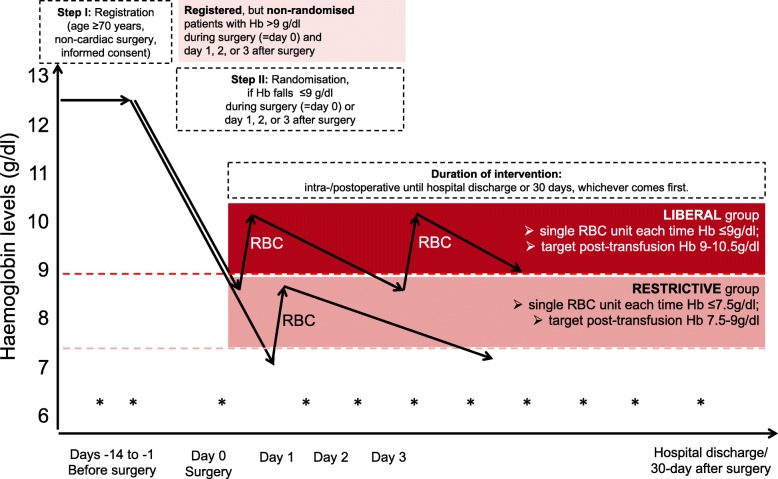
Study intervention. Hb levels will be determined from blood samples (*) mainly as part of the patient’s usual care at any time during or after surgery (up to 30 days after surgery; at least every 3 days), and after each transfused unit. Consenting patients will be registered (Step I) and will be randomised as soon as Hb falls ≤9 g/dl during surgery (=day 0) or day 1, 2, or 3 after surgery. Physicians will be instructed to transfuse RBC units each time Hb is lower the defined threshold and as soon as possible. The target post-transfusion Hb level needs to be reached within 24 h upon receipt of lab result at latest. The intervention per patient will be followed until hospital discharge or up to 30 days, whichever occurred first, comparable to recent large trials [5–7, 15]. In case of any massive or life-threatening bleeding, the single-unit policy should be paused

A two-step approach will be used (Figs. 1 and 2): In step I, elderly patients (age ≥ 70 years) will be registered if scheduled for elective, non-cardiac surgery of intermediate or high risk. Written informed consent is obtained from the patients themselves or from their legally authorised representative/legal guardian.

Risk assessment follows ESC/ESA guidelines: surgery-related risk of cardiovascular death and myocardial infarction [20]. Intermediate risk surgery (30-day risk 1–5%) includes e.g. intraperitoneal surgery (splenectomy, hiatal hernia), peripheral arterial angioplasty, endovascular aneurysm repair, as well as head and neck, major neurological/orthopaedic (hip and spine), major urological, major gynaecological, intra-thoracic surgery. High risk surgery (30-day risk > 5%) includes e.g. aortic and major vascular, open limb revascularisation, duodeno-pancreatic, liver resection, oesophagectomy, adrenal resection, total cystectomy.

Registered patients will be randomised later only if and as soon as Hb reaches ≤9 g/dl during surgery (=day 0) or day 1, 2, or 3 after surgery (step II). If Hb remains > 9 g/dl patients do not enter the main study but vital status (all-cause mortality) will be determined 90 days after surgery. Mild drop of haemoglobin < 9 g/dl due to induction of anaesthesia before skin incision is permitted and not an exclusion criteria.

Exclusion criteria at registration comprise preoperative severe anaemia with Hb level < 9 g/dl, chronic kidney injury requiring dialysis, suspected lack of compliance with follow-up procedures, expected death within 3 months, participation in other interventional trials or previous participation in our trial, temporary inability to provide informed consent with absence of a legally authorised representative/legal guardian, and preoperative autologous blood donation. Additional exclusion criteria at randomisation exclude patients who experienced any component of the composite endpoint or received any allogeneic blood transfusion since registration.

### Randomisation and Intervention

If Hb intra- or postoperatively reaches ≤9 g/dl (despite possible autologous transfusion) determined from blood samples mainly as part of the patient’s standard care at any time during surgery (=day 0) or day 1, 2, or 3 after surgery, registered patients will be randomised either to

- **LIBERAL group:** patients receive a single RBC unit each time Hb reaches ≤9 g/dl (≤ 5.6 mmol/l) with a target range for the post-transfusion Hb level of 9–10.5 g/dl (5.6–6.5 mmol/l), or
- **RESTRICTIVE group:** patients receive a single RBC unit each time Hb reaches ≤7.5 g/dl (≤ 4.7 mmol/l) with a target range for the post-transfusion Hb level of 7.5–9 g/dl (4.7–5.6 mmol/l).

The target post-transfusion Hb level needs to be reached within 24 h upon receipt of triggering lab result. The intervention per patient will be followed until hospital discharge or up to 30 days after surgery, whichever occurred first. Physicians will be allowed to refuse to transfuse, or transfuse patients irrespective of the group assignment in exceptional cases, e.g. hypervolaemia, symptomatic anaemia with physiological triggers of anaemic hypoxia, or massive/life-threatening bleeding, but must document the reason(s) in the study eCRF.

Central internet randomisation at the Clinical Trial Centre Leipzig uses block randomisation with variable block length stratified by centre. For urgent randomisation or in case of internet unavailability randomisation can be performed using sealed envelopes. Envelope randomisations will be checked during on-site monitoring.

All trial patients will receive standard perioperative care. The patients in both groups will not be exposed to additional risk since transfusion-strategy studies almost showed the same results. Hb transfusion thresholds used in previous studies varied from 7 to 10 g/dl for the restrictive and from 9 to 13 g/dl for the liberal group, respectively [5, 6, 15]. In addition RBC transfusion is the main treatment option for anaemia due to surgical blood loss. All patients benefit from intensive monitoring and consequently early detection of any decrease of Hb-level.

### Trial Drug

The strategies under evaluation use different Hb levels as trigger for RBC transfusions and aim at different target ranges of Hb levels to be maintained. The trial drug will be manufactured and used as in standard care. Only commercially available approved RBC units will be used within this clinical trial and a list of these approved RBCs is online available (http://www.pei.de/DE/arzneimittel/blutprodukte/blutkomponenten-zur-transfusion/erythrozytenkonzentrate/erythrozytenkonzentrate-node.html). RBCs are provided by the local blood bank according to clinical routine considering the requirements of § 63i AMG. This will assure the participants’ safety, the traceability and identification of the RBCs given. Therefore, a special labelling of the RBCs for the trial according to § 42 AMG and § 5 GCP-V is not necessary.

### Endpoints

The primary efficacy outcome is a binary composite of death from any cause and anaemia-associated, ischaemic events (defined as acute myocardial infarction, acute stroke, acute kidney injury stage III, acute mesenteric ischaemia, acute peripheral vascular ischaemia) within 90 days after surgery. With the proposed composite, we assess relevant anaemia-associated ischaemic events encompassing five different organs (brain, heart, kidney, gut, limbs) where the assigned Hb level/transfusion strategy will likely have an effect.

The primary efficacy outcome is defined as a composite of (within 90 days after surgery):

I. **All-cause mortality** is defined as death from any cause.
II. **Acute myocardial infarction** confirmed by a cardiologist.
III. **Acute ischaemic stroke** confirmed by a neurologist.
IV. **Acute kidney injury (stage III)** is defined according to the Kidney Disease Improving Global Outcomes criteria: Increase of plasma creatinine level ≥ 3 times within a time window of 7 days or initiation of renal replacement therapy [21].

(Serum creatinine concentration will be measured at least every 7 days until hospital discharge. Urine output criteria will not be used to define acute kidney injury because most of hospital do not mandate hourly urine output measurements on all patients, and because of the likelihood of inaccurate measurement in the substantial number of patients without urinary catheters.)

V. **Acute mesenteric ischaemia** is defined as ischaemia confirmed by intervention (abdominal surgery or mesenteric angiography).
VI. **Acute peripheral vascular ischaemia** is defined as a new non-thrombotic compromised circulation in a limb confirmed by angiography and/or leading to surgery.

After hospital discharge, events will only be considered as present if they lead to hospital re-admission or death. Direct transfer to another hospital will not be defined as re-admission.

Infection was not included into the composite endpoint since more transfusions may reduce ischaemic events, but might show a counter effect increasing the infection risk.

### Secondary endpoints

**Secondary outcome** measures are the following:

- The occurrence of any individual component of the composite at hospital discharge, at 90 days, and 1 year after surgery.
- Proportion of patients receiving RBC transfusion and the number of units transfused.
- Total length of stay in the intensive care unit and in hospital from randomisation to discharge (for strategy comparison); in addition, total length of stay in the intensive care unit and in hospital from admission to discharge will be used for descriptive purposes.
- The occurrence of acute kidney injury (stage I or II) defined according to the Kidney Disease Improving Global Outcomes criteria [21] (stage I: increase of plasma creatinine level ≥ 1.5–1.9 times baseline or ≥ 0.3 mg/dl within 48 h; stage II: increase of plasma creatinine level ≥ 2–2.9 times baseline within a time window of 7 days) during the initial hospital stay.
- Time to (first) infection (infection requiring therapeutic intravenous antibiotic treatment (pneumonia, wound infection, sepsis, central line associated blood stream infection [21–23])) during the initial hospital stay or leading to hospital re-admission within 90 days after surgery.
- Time to (first) re-hospitalisation within 90 days.
- Functional status (assessed by Barthel Index [24, 25] by telephone questionnaire).
- Health-related quality of life (assessed by EuroQoL EQ-5D [26, 27] and 12-item World Health Organisation Disability Assessment Schedule WHODAS 2.0 [28] by telephone questionnaire).

### Sample size considerations

For the primary endpoint, we expect an overall composite complication rate (OCCR) of about 25% at 90 days after surgery. This guestimate is based on a subgroup of elderly patients included in a large observational study in the field of Patient Blood Management [29], which reported most of the endpoint components, and is also based on the assumption that about 25–40% of registered patients will be randomised.

The effect size to be detected is set to an odds ratio of OR = 0.765. The available evidence on treatment differences from randomised trials concerning the old age group is sparse and inconsistent. Therefore, we choose an effect size, which is relevant from a clinical perspective. Assuming an OCCR of 25%, an odds ratio of OR = 0.765, corresponding to a 5% reduction in OCCR from 27.5% to 22.5% or risk reduction of 18%, would justify switching to the liberal transfusion strategy.

The target sample size is *N* = 2470 randomised patients. Randomisation of 2 × 1176 = 2352 patients is required using a two-sided significance level of 5% and requiring power of 80% for a test of the null-hypothesis OR = 1 versus OR = 0.765 based on the normal approximation to log (OR). We allow for a dropout rate of up to 5%.

### Statistical analysis

The full analysis set for the primary efficacy analysis will be as close as possible to the ideal implied by the intention-to-treat principle and includes all randomised subjects. A secondary per-protocol analysis of the primary outcome will be performed in all patients without major protocol violations.

Most endpoints will be analysed with scale-appropriate generalised linear mixed models adjusting for age, cancer surgery (y/n), type of surgery (intermediate- or high-risk), and incorporating centres as random effect. Further baseline clinical variables may be entered in secondary analyses.

For the primary endpoint, logistic regression will be used to estimate the treatment effect on the odds ratio scale with two-sided 95% confidence intervals provided. Secondarily, also point estimates and confidence intervals for the rate difference and the relative risk will be provided. The test of the null hypothesis that the odds ratio concerning the composite endpoint is equal to one will be tested using the Wald statistic for the coefficient of the treatment effect in the logistic regression.

### Interim analyses

One formal unblinded interim analysis is scheduled after about 1450 patients with 90-day endpoint information in order to detect early superiority. This interim analysis will use a significance level of alpha = 0.001 such that the final analysis does not require adjusting for multiple testing. With this interim analysis, we will have 80% power to detect an odds ratio of 0.6, which corresponds to an OCCR difference in the order of 10%.

If the interim analysis turns out significant, the trial will be stopped, unless the Data Monitoring Safety Board (DMSB) recommends otherwise. The responsible study biometrician will perform the formal interim analysis, write a strictly confidential report and discuss the results exclusively with the Data Monitoring Safety Board. If the DSMC recommends continuing with the trial, the Sponsor, the steering committee, the investigators, and the study team will only receive the information that the interim analysis for early superiority was performed and discussed with the DSMC and that the trial continues. All respective documents and analysis scripts are kept on a dedicated file system to which only the biometrician and his assistant have access rights.

### Planned exploratory sub-group analyses

We hypothesise that the benefit from a liberal transfusion strategy increases with declining anaemia compensatory capacity.

We will therefore perform exploratory subgroup analyses by

- Age (< 80 versus ≥80 years),
- Gender (male/female),
- American Society of Anaesthesiology Physical Status classification [30],
- presence of cancer (y/n),
- ischaemic heart disease (y/n),
- heart failure (y/n),
- peripheral vascular disease (y/n),
- previous stroke (y/n),

Any resulting hypothesis requires confirmation in independent data.

### Analysis in the larger set of all registered patients

We collect course of Hb levels during surgery (= day 0) or day 1, 2, 3 after surgery and the vital status at day 90 (all-cause mortality) for all registered patients.

The prognostic value of delta Hb (difference pre-op Hb and Hb on day three) and Barthel index on short term mortality will be analysed adding these factors to the generalised linear mixed model specified above.

### Clinical study monitoring

On-site monitoring follows a risk-adapted approach [31, 32]. Pre-study, initiation, regular, and closeout visits will be performed in all centres. The first regular visit takes place after discharge or day 30 after surgery of the first 3 randomized patients, the following visits will be performed after the discharge of additionally 15 more patients, but at least once a year. The intention of the visits will be to achieve high protocol compliance and data quality, as well as to ensure patients’ safety and rights.

Central monitoring will include a timely query management process based on consistency and plausibility checks combined with a dunning process for missing documentation. In addition, the trial biometrician performs statistical monitoring regularly in order to detect general and centre-specific problems in key study processes. Quality endpoints include accrual rate, rate of dropouts and protocol violations, promptness of randomisation when anaemia emerges, adherence to the assigned transfusion strategies, as well as timeliness of documentation.

## Discussion

The transfusion of allogenic RBC units is the standard procedure to treat peri- and postoperative severe anaemia. More than 50% of all RBC transfusions are used in old and frail patients, and current population dynamics in most developed countries will lead to an increasing demand for RBC transfusions [19]. Elderly patients have an increased prevalence of cardiovascular comorbidities and decline of functional reserves. Therefore, anaemia-related compensatory mechanisms are severely impaired in elderly patients, which may result in greater vulnerability to anaemia-related ischaemic events and perioperative complications [11, 12].

Here, the LIBERAL-Trial will provide a definite answer for this patient group whether a liberal transfusion strategy reduces the occurrence of major adverse events defined as the composite of all-cause mortality and severe ischaemic events within 90 days after non-cardiac surgery compared to a restrictive strategy.

Potential limitations might refer to cross-over between groups, needing to ‘register’ a large number of patients, lack of recruiting, and drop-out due to various protocol violations.

## Trial Status

Study protocol (V3.0) was finalised at 17th of October 2017. This trial was registered at https://clinicaltrials.gov/ct2/show/NCT03369210 (identifier: NCT03369210) at 12th of November 2017. Recruitment and enrolment of the patients started in January 2018, more than 200 patients have been registered and more than 100 randomised so far. The planned recruitment lasts 36 months.

## Additional file

Additional file 1: SPIRIT 2013 Checklist. Recommended items to address in a clinical trial protocol and related documents*. (DOCX 52 kb)

## Abbreviations

AMG: Arzneimittelgesetz
BGA: blood gas analysis
eCRF: electronic case report form
GCP: Good clinical practice
Hb: haemoglobin
OCCR: overall composite complication rate
RBC: red blood cell

## References

[1] Carson JL, Guyatt G, Heddle NM, Grossman BJ, Cohn CS, Fung MK (2016). Clinical Practice Guidelines From the AABB: Red Blood Cell Transfusion Thresholds and Storage. JAMA.

[2] Padhi S, Kemmis-Betty S, Rajesh S, Hill J, Murphy MF, Guideline Development G (2015). Blood transfusion: summary of NICE guidance. BMJ.

[3] Carson JL, Sieber F, Cook DR, Hoover DR, Noveck H, Chaitman BR (2015). Liberal versus restrictive blood transfusion strategy: 3-year survival and cause of death results from the FOCUS randomised controlled trial. Lancet.

[4] Hebert PC, Wells G, Blajchman MA, Marshall J, Martin C, Pagliarello G (1999). A multicenter, randomized, controlled clinical trial of transfusion requirements in critical care. Transfusion Requirements in Critical Care Investigators, Canadian Critical Care Trials Group. N Engl J Med.

[5] Holst LB, Haase N, Wetterslev J, Wernerman J, Guttormsen AB, Karlsson S (2014). Lower versus higher hemoglobin threshold for transfusion in septic shock. N Engl J Med.

[6] Murphy GJ, Pike K, Rogers CA, Wordsworth S, Stokes EA, Angelini GD (2015). Liberal or restrictive transfusion after cardiac surgery. N Engl J Med.

[7] Villanueva C, Colomo A, Bosch A, Concepcion M, Hernandez-Gea V, Aracil C (2013). Transfusion strategies for acute upper gastrointestinal bleeding. N Engl J Med.

[8] Neuhauser HK, Adler C, Rosario AS, Diederichs C, Ellert U (2015). Hypertension prevalence, awareness, treatment and control in Germany 1998 and 2008-11. J Hum Hypertens.

[9] Sue Kirkman M, Briscoe VJ, Clark N, Florez H, Haas LB, Halter JB (2012). Diabetes in older adults: a consensus report. J Am Geriatr Soc.

[10] Lubitz SA, Bauer KA, Benjamin EJ, Besdine RW, Forman DE, Gurol ME (2013). Stroke prevention in atrial fibrillation in older adults: existing knowledge gaps and areas for innovation: a summary of an American Federation for Aging research seminar. J Am Geriatr Soc.

[11] Goodnough LT, Schrier SL (2014). Evaluation and management of anemia in the elderly. Am J Hematol.

[12] Fried LP, Tangen CM, Walston J, Newman AB, Hirsch C, Gottdiener J (2001). Frailty in older adults: evidence for a phenotype. J Gerontol A Biol Sci Med Sci.

[13] Carson JL, Brooks MM, Abbott JD, Chaitman B, Kelsey SF, Triulzi DJ (2013). Liberal versus restrictive transfusion thresholds for patients with symptomatic coronary artery disease. Am Heart J.

[14] Carson JL, Terrin ML, Barton FB, Aaron R, Greenburg AG, Heck DA (1998). A pilot randomized trial comparing symptomatic vs. hemoglobin-level-driven red blood cell transfusions following hip fracture. Transfusion.

[15] Carson JL, Terrin ML, Noveck H, Sanders DW, Chaitman BR, Rhoads GG (2011). Liberal or restrictive transfusion in high-risk patients after hip surgery. N Engl J Med.

[16] Simon GI, Craswell A, Thom O, Fung YL (2017). Outcomes of restrictive versus liberal transfusion strategies in older adults from nine randomised controlled trials: a systematic review and meta-analysis. Lancet Haematol.

[17] Frank SM, Savage WJ, Rothschild JA, Rivers RJ, Ness PM, Paul SL (2012). Variability in blood and blood component utilization as assessed by an anesthesia information management system. Anesthesiology.

[18] Gombotz H, Rehak PH, Shander A, Hofmann A (2014). The second Austrian benchmark study for blood use in elective surgery: results and practice change. Transfusion.

[19] Greinacher A, Fendrich K, Brzenska R, Kiefel V, Hoffmann W (2011). Implications of demographics on future blood supply: a population-based cross-sectional study. Transfusion.

[20] Kristensen SD, Knuuti J, Saraste A, Anker S, Botker HE, Authors/Task Force M (2014). ESC/ESA Guidelines on non-cardiac surgery: cardiovascular assessment and management: The Joint Task Force on non-cardiac surgery: cardiovascular assessment and management of the European Society of Cardiology (ESC) and the European Society of Anaesthesiology (ESA). Eur Heart J.

[21] Jammer I, Wickboldt N, Sander M, Smith A, Schultz MJ, Pelosi P (2015). Standards for definitions and use of outcome measures for clinical effectiveness research in perioperative medicine: European Perioperative Clinical Outcome (EPCO) definitions: a statement from the ESA-ESICM joint taskforce on perioperative outcome measures. Eur J Anaesthesiol.

[22] Dellinger RP, Levy MM, Rhodes A, Annane D, Gerlach H, Opal SM (2013). Surviving Sepsis Campaign: international guidelines for management of severe sepsis and septic shock, 2012. Intensive Care Med.

[23] Rohde JM, Dimcheff DE, Blumberg N, Saint S, Langa KM, Kuhn L (2014). Health care-associated infection after red blood cell transfusion: a systematic review and meta-analysis. JAMA.

[24] Collin C, Wade DT, Davies S, Horne V (1988). The Barthel ADL Index: a reliability study. Int Disabil Stud.

[25] Barthel Index Hamburger Manual [https://www.dimdi.de/static/.downloads/deutsch/hamburger-manual-nov2004.pdf]. Accessed 29 Jan 2019.

[26] Rabin R, de Charro F (2001). EQ-5D: a measure of health status from the EuroQol Group. Ann Med.

[27] Tidermark J, Bergstrom G (2007). Responsiveness of the EuroQol (EQ-5D) and the Nottingham Health Profile (NHP) in elderly patients with femoral neck fractures. Qual Life Res.

[28] Xenouli G, Xenoulis K, Sarafis P, Niakas D, Alexopoulos EC (2016). Validation of the World Health Organization Disability Assessment Schedule (WHO-DAS II) in Greek and its added value to the Short Form 36 (SF-36) in a sample of people with or without disabilities. Disabil Health J.

[29] Meybohm P, Herrmann E, Steinbicker AU, Wittmann M, Gruenewald M, Fischer D (2016). Patient Blood Management is Associated With a Substantial Reduction of Red Blood Cell Utilization and Safe for Patient’s Outcome. A Prospective, Multicenter Cohort Study With a Noninferiority Design. Ann Surg.

[30] Hackett NJ, De Oliveira GS, Jain UK, Kim JY. ASA class is a reliable independent predictor of medical complications and mortality following surgery. Int J Surg. Int J Surg. 2015;18:184-90. 10.1016/j.ijsu.2015.04.079 25937154

[31] Brosteanu O, Houben P, Ihrig K, Ohmann C, Paulus U, Pfistner B (2009). Risk analysis and risk adapted on-site monitoring in noncommercial clinical trials. Clin Trials.

[32] Brosteanu O, Schwarz G, Houben P, Paulus U, Strenge-Hesse A, Zettelmeyer U (2017). Risk-adapted monitoring is not inferior to extensive on-site monitoring: Results of the ADAMON cluster-randomised study. Clin Trials.

